# Childhood epilepsies

**Published:** 2017-01

**Authors:** Ali H. Alwadei

**Affiliations:** *From the Department of Pediatric Neurology, National Neuroscience Institute, King Fahad Medical City, Riyadh, Kingdom of Saudi Arabia*

The Neurosciences Journal includes this section of multiple choice questions as part of its commitment to continuous education and learning in Neurosciences. Experts in various neuroscience specialties are invited to participate with their knowledge and expertise in this section.

Neurology, neurosurgery, and other board residents are encouraged to read this section to improve their knowledge and direct their reading for written examinations.

**Choose the most appropriate single answer**.

1. Symptomatogenic zone is defined as:
  - Area of cortex that initiates or generates seizures.
  - Cortical area producing non-epileptic dysfunction.
  - Portion of the brain that produces the first clinical symptoms.
  - Total area of brain that is necessary to generate seizures and that must be removed to abolish seizures.
2. Saleh is a 2-year-old male who presented with a self-aborted generalized tonic clonic seizure, which lasted for few minutes with high-grade fever due to pharyngitis. His father who is now a mathematics teacher, used to have seizures with fever between ages 2-4 years. Saleh’s mother is asking whether her child is going to be epileptic in future or not. Best response is that you would tell her that Saleh’s risk of future epilepsy is
  - 2-4% which is at least double the risk of that in normal population.
  - The probability of not developing epilepsy is 96-98%.
  - 30% chance that he becomes epileptic.
  - There is no way to predict future risk.
3. You are evaluating a 10-year-old developmentally appropriate boy in your clinic for 2 episodes of protracted confusion. Father claimed that, his head was shaking from side to side and he was partially responsive during the 2 episodes. One of the 2 episodes lasted for more than 15 hours. His 35-year-old uncle is known to have epilepsy diagnosed at age 15 years and he is fairly controlled on valproic acid. Next step is:
  - Arrange a non-urgent EEG in next few months.
  - Ask the child to blow up a napkin for 3-5 minutes.
  - Send the child for an MRI-Brain
  - Thorough physical/neurological examination.
4. Loading dose of Antiepileptic Drug in neonates and young infants is:
  - Larger than that required by an adult due to decreased Vd (Volume of distribution).
  - Larger than that required by an adult due to an increased Vd.
  - Not any different than that required by an adult.
  - Smaller than that required by an adult due to an increased Vd.
5. An 8-year-old boy with complex partial seizures since age 1½ years. MRI Brain disclosed a cystic space-occupying lesion in the right hippocampus and parahippocampal gyrus with surrounding areas of hyperintensity as seen in the images. This is suggestive of:
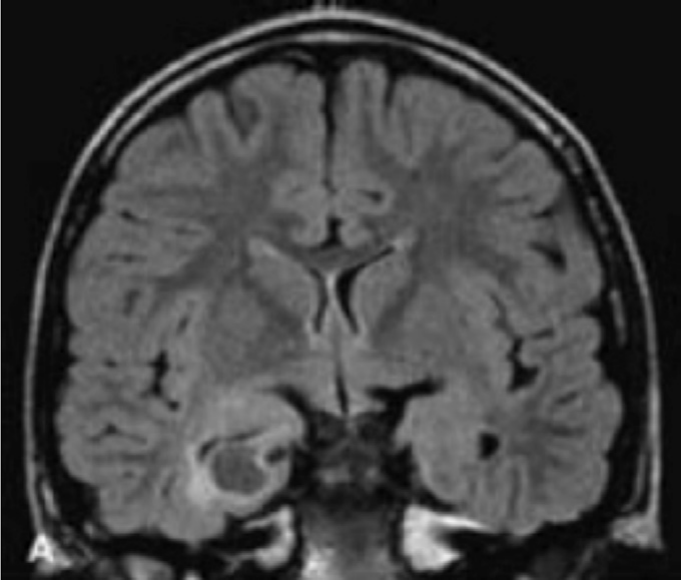
  - Dysembryoplastic neuroepithelial tumor.
  - Ganglioglioma.
  - Mesial Temporal Sclerosis.
  - Tuberculoma.

**Answers:**

1. **c**
  In symptomatic epilepsies, the epileptogenic lesion is the pathologic substrate for the epilepsy; it can usually be identified on MRI, although EEG remains necessary to demonstrate epileptogenicity of a lesion. Seizures can arise within, adjacent to, or even sometimes distant from an epileptogenic lesion. The symptomatogenic zone is that portion of the brain responsible for producing the first clinical ictal symptoms or signs, whereas the functional deficit zone is the cortical area or areas exhibiting focal nonepileptic dysfunction. Finally, the epileptogenic zone is the total area of brain that is necessary and sufficient to generate seizures and that must be removed to abolish seizures. The fact that the epileptogenic zone cannot be defined with precision accounts for the lack of a uniformly successful outcome following resective surgery for focal seizures. The problem is greater in extratemporal than in temporal lobe epilepsy.^1^,^2^
2. **b**
  A is still correct but it is not the “best” way of communicating the answer. The incidence of afebrile seizures after febrile convulsions at 10 years of age is 2.5%. The cumulative risk of an unprovoked seizure increases with age.^3^-^5^
3. **b**
  Hyperventilation may disclose the diagnosis which is absence status. Absence status epilepticus is a prolonged, generalized absence seizure that usually lasts for hours and can even last for days. The cardinal symptom is the altered state of consciousness while the patient is usually fully alert and partially responsive. Absence status epilepticus may be typical, occurring in patients with idiopathic generalized epilepsy, or atypical, occurring in patients with neurocognitive impairment as with epileptic encephalopathies. Absence status epilepticus may also appear de novo, mainly in adults without a previous history of epilepsy. Absence status epilepticus is often misdiagnosed as focal status epilepticus or a confusional nonepileptic condition or epileptic prodrome. Frequently, absence status epilepticus occurs because of ill-advised antiepileptic drug treatment, such as with tiagabine or carbamazepine in patients with idiopathic generalized epilepsy.^6^
4. **b**
  Determining the right dose for drugs used to treat neonates is critically important. Neonates have significant differences in physiology affecting drug absorption, distribution, metabolism, and elimination that make extrapolating dosages from adults and older children inappropriate.^7^,^8^
5. **b**
  The patient was seizure free for several months following anterior temporal lobectomy with amygdalohippocampectomy. Ganglioglioma as a neoplasm composed of neoplastic neural and neoplastic glial cells. Ganglioglioma may occur in any part of the neuraxis, but the most frequent site is the temporal lobe. Ganglioglioma is a common cause of tumor-related refractory epilepsies and in some series account for nearly half of the tumoral substrates, though in others it is lower.^1^
